# Neurophysiologic Features Reflecting Brain Injury During Pediatric ECMO Support

**DOI:** 10.1007/s12028-023-01836-9

**Published:** 2023-09-11

**Authors:** Damla Hanalioglu, M. ’Hamed Temkit, Kara Hildebrandt, Elizabeth MackDiaz, Zachary Goldstein, Shefali Aggarwal, Brian Appavu

**Affiliations:** 1 Division of Neurology, Department of Neuroscience, Barrow Neurological Institute at Phoenix Children’s Hospital, 1919 E Thomas Rd, Phoenix, AZ 85016 USA; 2 Department of Pediatrics, Medical University of South Carolina, Charleston, SC USA; 3 Department of Child Health, The University of Arizona College of Medicine – Phoenix, Phoenix, AZ USA

**Keywords:** Extracorporeal membrane oxygenation, Electroencephalography, Transcranial Doppler ultrasound, Seizures, Acute brain injury

## Abstract

**Background:**

Extracorporeal membrane oxygenation (ECMO) provides lifesaving support to critically ill patients who experience refractory cardiopulmonary failure but carries a high risk for acute brain injury. We aimed to identify characteristics reflecting acute brain injury in children requiring ECMO support.

**Methods:**

This is a prospective observational study from 2019 to 2022 of pediatric ECMO patients undergoing neuromonitoring, including continuous electroencephalography, cerebral oximetry, and transcranial Doppler ultrasound (TCD). The primary outcome was acute brain injury. Clinical and neuromonitoring characteristics were collected. Multivariate logistic regression was implemented to model odds ratios (ORs) and identify the combined characteristics that best discriminate risk of acute brain injury using the area under the receiver operating characteristic curve.

**Results:**

Seventy-five pediatric patients requiring ECMO support were enrolled in this study, and 62 underwent neuroimaging or autopsy evaluations. Of these 62 patients, 19 experienced acute brain injury (30.6%), including seven (36.8%) with arterial ischemic stroke, four (21.1%) with hemorrhagic stroke, seven with hypoxic-ischemic brain injury (36.8%), and one (5.3%) with both arterial ischemic stroke and hypoxic-ischemic brain injury. A univariate analysis demonstrated acute brain injury to be associated with maximum hourly seizure burden (*p* = 0.021), electroencephalographic suppression percentage (*p* = 0.022), increased interhemispheric differences in electroencephalographic total power (*p* = 0.023) and amplitude (*p* = 0.017), and increased differences in TCD Thrombolysis in Brain Ischemia (TIBI) scores between bilateral middle cerebral arteries (*p* = 0.023). Best subset model selection identified increased seizure burden (OR = 2.07, partial *R*^2^ = 0.48, *p* = 0.013), increased quantitative electroencephalographic interhemispheric amplitude differences (OR = 2.41, partial *R*^2^ = 0.48, *p* = 0.013), and increased interhemispheric TCD TIBI score differences (OR = 4.66, partial *R*^2^ = 0.49, *p* = 0.006) to be independently associated with acute brain injury (area under the receiver operating characteristic curve = 0.92).

**Conclusions:**

Increased seizure burden and increased interhemispheric differences in both quantitative electroencephalographic amplitude and TCD MCA TIBI scores are independently associated with acute brain injury in children undergoing ECMO support.

**Supplementary Information:**

The online version contains supplementary material available at 10.1007/s12028-023-01836-9.

## Introduction

Extracorporeal membrane oxygenation (ECMO) provides life-saving support to critically ill patients who experience refractory cardiopulmonary failure [1]. The utility of ECMO has increased worldwide [2], and despite clear benefit, its use is associated with significant morbidity commonly attributed to acute brain injury [3–6]. Timely and accurate recognition of acute brain injury in this population is necessary to mitigate secondary brain injury and improve outcomes. However, frequent use of sedation and neuromuscular blockade limit use of neurologic examinations to detect acute brain injury, and neuroimaging is challenging in the setting of hemodynamic instability and transport challenges [7]. Bedside neuromonitoring may be useful in recognition of acute brain injury, which can aid clinicians in management toward either preventing primary brain injury or mitigating resultant secondary brain injury.

Multimodality neuromonitoring (MMM) allows for time-synchronized and time-integrated collection and analysis of high-frequency physiologic data [8, 9]. Retrospective studies investigating the utility of continuous electroencephalography (cEEG), transcranial Doppler ultrasound (TCD), and cerebral regional oximetry (rSO_2_) and their correlations with continuous physiologic parameters in ECMO patients have found associations between altered physiologic parameters and acute brain injury as well as patient outcomes [10–21]. However, these studies have looked at these neuromonitoring tools in isolation without comparison of each other, with fewer studies describing usefulness or feasibility of combined and integrated MMM in detecting acute brain injury of pediatric ECMO patients in a prospective manner [15, 22]. The primary objective of this study is to prospectively identify biomarkers of acute brain injury using MMM, clinical, and ECMO circuit characteristics in children during ECMO support.

## Methods

### Study Design, Setting, and Participants

This study is a single-center prospective observational study conducted at the Phoenix Children’s Hospital neonatal, pediatric, and cardiovascular intensive care units (ICUs) that enrolled consecutive ECMO patients aged 0–21 years who underwent MMM from June 2019 to April 2022.

Inclusion criteria included children from 0 to 21 years of age who required ECMO support. Patients were excluded in case of prior known acquired brain injury, sickle cell disease, and Moya-Moya disease. Patients with congenital brain malformations were not excluded, although none were identified with such malformations during neuroimaging. This study was approved by the Phoenix Children’s Hospital Institutional Review Board (No. 19-257; approval date 05/16/2019; institutional review board study title: Multivariate Prediction of Stroke in Children Requiring Mechanical Circulatory Support). All procedures were followed in accordance with the ethical standards of the Institutional Review Board at Phoenix Children’s Hospital. Written informed consent was obtained from the parents or legal guardians for each participant, with consent sought within the first 24 h of ECMO cannulation.

### Outcome Measures, Data Sources, and Data Collection

The primary outcome was the presence of acute brain injury during ECMO support from either neuroradiographic imaging or autopsy findings. All patients were managed according to up-to-date clinical practice guidelines [23, 24]. As standard of care, all patients underwent serial neurological examinations, continuous rSO_2_, daily head ultrasound imaging for the first 5 days of monitoring if age appropriate and as requested, and head computed tomography (CT) when clinical concerns for neurologic changes occurred. Neurologic examinations were attempted in the morning each day, and continuous rSO_2_ remained continuous throughout the monitoring session. Surviving patients underwent brain magnetic resonance imaging (MRI) after decannulation, if able. The diagnosis of acute brain injury was made based on neuroanatomic evidence of arterial ischemic stroke (AIS), hemorrhagic stroke (HS), or hypoxic-ischemic brain injury (HIBI) from brain magnetic resonance, CT, or head ultrasound images. We excluded microhemorrhages, extra-axial fluid collections, and white matter hyperintensities < 3 mm as biomarkers of acute brain injury. In case of a deceased patient without any neurologic imaging, findings compatible with acute brain injury on autopsy reports were used.

As part of this study, patients underwent MMM that included cEEG, cerebral rSO_2_, and daily TCD of the bilateral middle cerebral arteries (MCAs). cEEG and cerebral rSO_2_ were collected as part of institutional standard-of-care procedures during ECMO support, whereas daily TCD was performed as a new element to this research study. cEEG was monitored as standard of care for the initial 48 h of ECMO monitoring and for longer periods of time at the discretion of the clinical team. If cEEG monitoring was performed for 120 consecutive hours, institutional protocols recommended 48 h of scalp rest before cEEG could be resumed. MMM and systemic hemodynamic monitoring data were integrated through a MMM device (CNS200; Moberg ICU Solutions, Philadelphia, PA). Intensive Care Monitor Plus (ICM+) software (Cambridge, UK) was used to visualize and process all MMM data and calculate model-based indices of cerebrovascular pressure reactivity (CVPR) and autonomic function. Time-series physiologic data were collected from ECMO initiation to decannulation in patients without brain injury or with brain injury detected from autopsy evaluations and from ECMO initiation to detection of brain injury on neuroimaging for relevant patients. Data with substantial artifacts observed through visual analysis were removed.

Continuous electroencephalography (cEEG) data were captured using institutional clinical hardware (Xltek; Natus Medical, Pleasanton, CA) under the International 10–20 system. Collected electroencephalography (EEG) characteristics included maximum hourly electrographic seizure burden, presence of interictal epileptiform discharges, and quantitative EEG (qEEG) features, such as amplitude; alpha (8–13 Hz), beta (13–20 Hz), theta (4–7 Hz), and delta power (0–4 Hz); total power (1–20 Hz); alpha-delta power ratio; and suppression percentage (SP). qEEG analysis was performed using Persyst Advanced Review (Persyst, Prescott, AZ). Amplitude was computed in microvolts from the Persyst standard processing engine, which evaluates the average amplitude in each epoch based on absolute value of baseline-to-peak amplitude. SP represents a running average of percentage of EEG activity that appears suppressed; values approaching 0 represent no suppression, and values approaching 100 represent full suppression. Our SP represents the calculated suppression ratio produced from the Persyst standard processing engine, which evaluates 10-s epochs and estimates the total duration of the epoch in which EEG activity is < 3 µV and > 0.5 s. In nonneonates (> 1 month of conceptual age), interictal epileptiform discharges were characterized as spike or polyspike discharges or sharp waves. In neonates, interictal epileptiform discharges were characterized by spike or polyspike discharges or sharp waves that occurred with predominance over specific regions without a multifocal distribution. In neonates, multifocal sharp transients were not qualified as interictal epileptiform discharges. Interhemispheric differences in qEEG parameters were calculated by taking the absolute difference in each of the previously described qEEG parameters from EEG electrodes over the right hemisphere as compared to EEG electrodes over the left hemisphere. Electrographic seizures and maximal hourly seizure burden were defined as previously described, with quantification of seizures during each hourly interval and determination of the maximal seizure count within a 1-h epoch [25, 26]. Qualification of all EEG data was confirmed through visual inspection of raw EEG waveforms by a board-certified epileptologist (BA). Qualification of seizures and epileptiform discharges was initially detected by a board-certified epileptologist and later confirmed through standard clinical reports of the clinical epileptologist on service.

Patients underwent TCD evaluations of systolic, diastolic, and mean flow velocities in bilateral MCAs within 24 h of ECMO initiation and daily thereafter. Systolic velocity represents the peak blood flow velocity during the systolic phase of the cardiac cycle, diastolic velocity represents the lowest blood flow velocity during the diastolic phase of the cardiac cycle, and mean flow velocity represents the average blood flow velocity over the entire cardiac cycle. Mean flow velocity is calculated by integrating the instantaneous velocity measurements obtained throughout the systolic and diastolic phases of the cardiac cycle and dividing it by the total duration of cycles. We used power M-mode TCD machines (Spencer Technologies and Novasignal Lucid), which interface with the MMM device, facilitating time synchronization of TCD waveforms with other physiologic parameters. Transtemporal acoustic windows were identified by patient anatomic landmarks. Flow velocity measurements were collected every 2 mm along the entire course of the vessel, with maximal velocities collected. The pulsatility index (PI) was derived by the TCD unit for each set measurements according to the following equation: PI = (systolic velocity − diastolic velocity)/mean flow velocity. TCD data, either on the last day of ECMO support (if no neuroimaging of brain injury) or just prior to detection of brain injury on neuroimaging, were incorporated into acute brain injury detection models to assess findings that would be most reflective of acute brain injury either just before or during the ECMO course. All TCD measurements were evaluated by a board-certified neurosonologist, and waveform grading was based on the Thrombolysis in Brain Ischemia (TIBI) scale [27] when evidence of MCA pulsatility was present on TCD examinations. If patients experienced nonpulsatile MCA flow on TCD, they were scored as having nonpulsatile flow.

Continuous cerebral and systemic hemodynamic physiology data, including arterial blood pressure (ABP), central venous pressure, rSO_2_, and heart rate, were acquired. ABP was continuously monitored from an indwelling radial or femoral catheter. Near-infrared spectroscopy sensors were placed on the bifrontal forehead, and rSO_2_ was continuously measured (Medtronic INVOS).

Cerebrovascular pressure reactivity (CVPR) was investigated using the cerebral oximetry index (COx) [28], which represents a moving Pearson correlation coefficient of ABP and rSO_2_. COx is calculated within a 5-min averaging window updated every 60 s. CO*x* values approaching 1 are postulated to represent inefficient CVPR, whereas values that are negative or approaching 0 are postulated to represent efficient CVPR. We used previously described methods to evaluate time spent below the lower limit of CVPR (LLA) and time spent above the upper limit of limit of CVPR (ULA) as well as optimal values of CVPR (ABPOpt) [28, 29]. ABPOpt values were identified using previously described methods of plotting ABP values with COx and identifying the minimum ABP value on a parabolic curve fit to the data with a multiwindow algorithm [28]. ABPOpt values were calculated from every 1 min from ABP and COx values taken from the moving 4-h window. Specific details regarding multiwindow algorithm calculation and criteria for value rejection and fitting have been previously described and are summarized in Supplement 1 [29]. LLA and ULA were determined for COx as the ABP values at which the curve exceeded a CO*x* value of 0.30, as previously described. LLA and ULA were determined as the lowest and highest ABP values with such thresholds, respectively. The percentage of time that ABP was below and above ABPOpt, LLA, and ULA were calculated for each patient during their ECMO course.

To evaluate for autonomic function, we investigated baroreflex sensitivity and three measures of heart rate variability: standard deviation of heart rate, root-mean-square of successive differences in heart rate, and low-frequency to high-frequency ratio. Methods of calculating these parameters have been previously described and are summarized in Supplement 2 [30].

In addition to continuous physiologic data, demographic data collected included age, sex, race, and indication for ECMO support. ECMO circuit characteristics investigated included type of circuit initiated (veno-arterial [VA] or veno-venous [VV]), arterial cannulation site (carotid vs. aorta vs. femoral), venous cannulation site (e.g., femoral vs. right atrial vs. internal jugular vein), arterial and venous catheter sizes, duration of ECMO course, and minimal and maximal anti-Xa levels, pump flow rates, sweep gas flow, and sweep gas inlet oxygen fraction. We also collected information regarding whether extracorporeal cardiopulmonary resuscitation was used prior to ECMO cannulation.

### Statistical Analysis

Descriptive characteristics were summarized by using median and interquartile range (IQR) or counts and percentages as appropriate. Univariate logistic regression modeling was used to identify risk factors or features that were associated with acute brain injury. The strength of univariate associations was summarized using odds ratios (ORs) and their corresponding 95% confidence intervals (CIs) and *p* values. All risk factors with *p* values less than 0.20 were entered in a multivariable logistic regression model using a guided stepwise method and the Akaike information criterion to identify combined characteristics best discriminating risk toward acute brain injury using the area under the receiver operating characteristic curve. In the multivariable model, the contribution of each predictor was summarized using the partial *R*^2^ for generalized linear models [31]. Univariate logistic regression was also applied to explore factors associated with AIS, HS, or HIBI. Statistical analyses were performed using the statistical software packages SAS 9.4 (SAS Institute, Inc., Cary, NC) and R Studio Version 3.4.1.

## Results

### Basic Characteristics of the Study Population

**Fig. 1:**
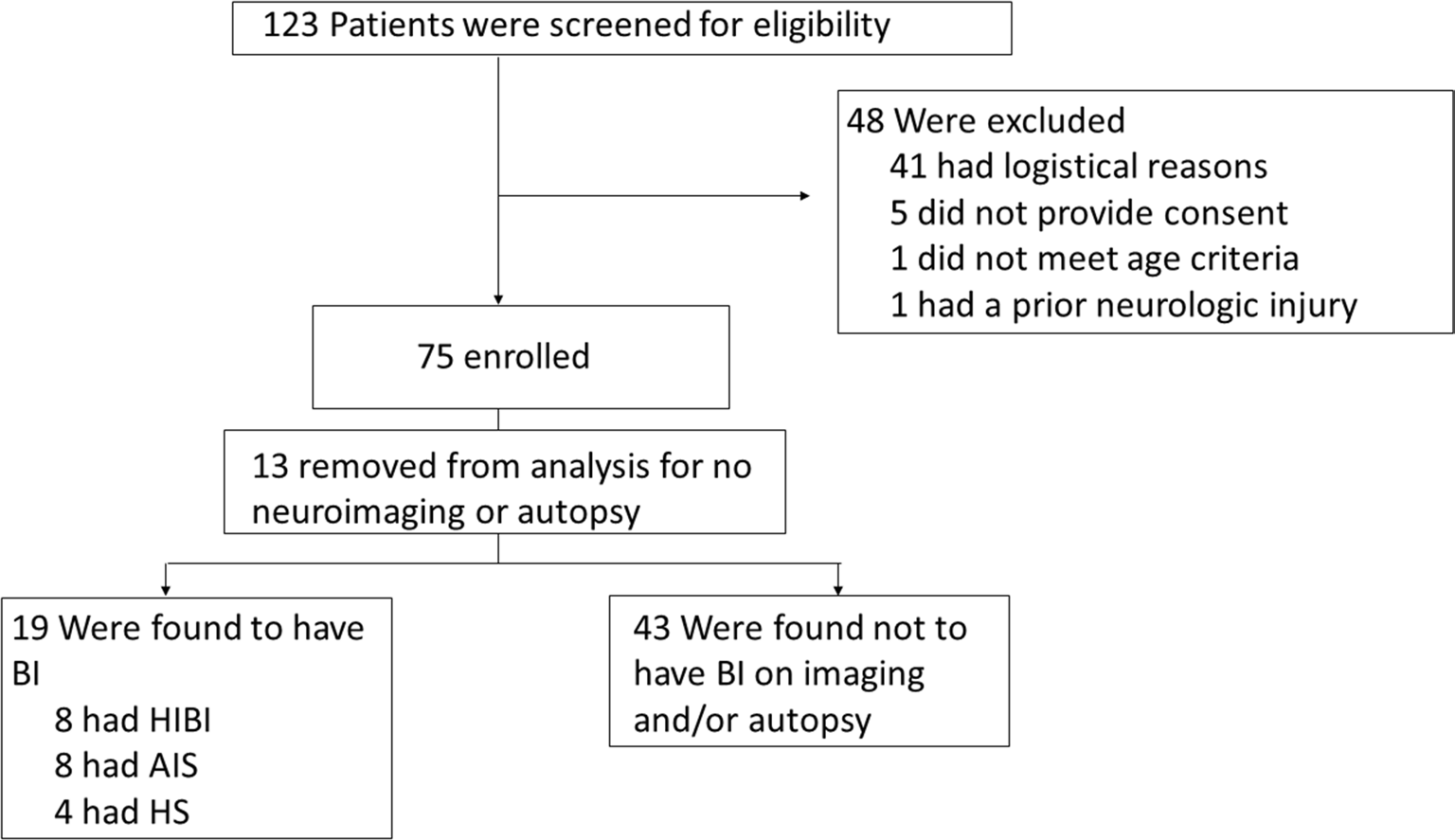
Patient enrollment. AIS arterial ischemic stroke, BI brain injury, HIBI hypoxic-ischemic brain injury, HS hemorrhagic stroke

Demographic data and ECMO circuit characteristics are summarized in Table 1, and enrollment is summarized in Fig. 1. One hundred twenty-three patients undergoing ECMO support were screened and identified from June 2019 to April 2022. Seventy-five such patients (61.0%) were prospectively enrolled and underwent MMM. One patient (1.0%) was determined ineligible because of age criteria, and one patient (1.0%) was deemed ineligible because of a prior AIS. Five patients (4.1%) did not provide consent. Consent was not obtained for 41 patients (33.3%) because of logistic reasons (see Supplement 3). To accurately assess for acute brain injury as the outcome, we excluded patients from analysis if they did not undergo neuroimaging or an autopsy after death, leaving 62 of the 75 (82.7%) enrolled patients available for analysis. The median age was 0.8 (IQR 0.1–7.2) years, and 28 patients (45.2%) were female. Common indications for ECMO included acute respiratory failure (33%) followed by cardiogenic shock (31%) and cardiac arrest (19%). Forty-six (74.2%) patients underwent VA-ECMO, fifteen (24.2%) underwent VV-ECMO, and one (1.6%) underwent VV-ECMO and later switched to VA-ECMO. Seventeen (27.5%) patients underwent extracorporeal cardiopulmonary resuscitation prior to ECMO cannulation, all of whom received VA-ECMO support. Arterial cannulation occurred in the right carotid artery for 41 patients (66.1%), in the aorta for 14 patients (22.6%), and in the femoral artery for seven patients (11.3%). Venous cannulation occurred in the internal jugular vein for 41 patients (66.1%), in the femoral vein for six patients (9.7%), and in the right atrium for 15 patients (24.2%). The median length of hospital stay was 35.5 (IQR 21.5–81.0) days. The median length of ECMO duration was 7.0 (IQR 3.3–13.5) days. During their ECMO course, patients were monitored on cEEG for a median of 50.0% of their course (IQR 29.2–100.0). The median time from ECMO cannulation to cEEG initiation was 5.0 h (IQR 3.0–7.8). The percentage of artifact on multimodality monitoring that was identified and removed during ECMO monitoring was a median of 16.5% (IQR 12.0–25.8).

**Table 1:** Patient characteristics and ECMO circuit data

Characteristic (*n* = 62)	*n*	%
Female sex	28	45.2
Ethnicity		
Hispanic	22	35.5
White	24	38.7
Native American	7	11.3
African American	7	11.3
Asian American	2	3.2
Acute brain injury	19	30.6
AIS	8	12.9
HS	4	6.5
HIBI	8	12.9
VA-ECMO	46	74.2
VV-ECMO	15	24.2
VV-ECMO to VA-ECMO	1	1.6
Arterial cannulation		
Right carotid artery	41	66.1
Aorta	14	22.6
Femoral artery	7	11.3
Venous cannulation		
Internal jugular vein	41	66.1
Right atrium	15	24.2
Femoral vein	6	9.7

	Median (IQR)	Range
Age (year)	0.8 (0.1, 7.2)	0–18
Arterial catheter size (mm)	10.0 (8.0, 15.8)	8–25
Venous catheter size (mm)	14.0 (12.0, 22.5)	8–31
Pump flow rate, maximal (L/min)	0.94 (0.51, 2.95)	0.30–5.50
Pump flow rate, minimal (L/min)	0.40 (0.24, 1.59)	0.00–3.97
Sweep gas flow (O_2_), maximal (L/min)	2.00 (0.50, 5.00)	0.30–14.0
Sweep gas flow (O_2_), minimal (L/min)	0.10 (0.10, 0.48)	0.00–8.00
VA-ECMO FsO_2_, maximal (%)	70.0 (60.0, 80.0)	40.0–100.0
VA-ECMO FsO_2_, minimal (%)	35.0 (21.0, 40.0)	0.0–90.0
VV-ECMO FsO_2_, maximal (%)	100.0 (100.0, 100.0)	70.0–100.0
VV-ECMO FsO_2_, minimal (%)	37.5 (0.8, 48.8)	0.0–75.0
ECMO duration (days)	7.0 (3.3, 13.5)	1.0–36.0
Hospital length (days)	35.5 (21.5, 81.0)	1.0–424.0

*AIS* arterial ischemic stroke, *ECMO* extracorporeal membrane oxygenation, *FsO*_*2*_ sweep gas inlet oxygen fraction, *HIBI* hypoxic-ischemic brain injury, *HS* hemorrhagic stroke, *IQR* interquartile range, *VA* veno-arterial, *VV* veno-venous

At least one neuroimaging modality was used in 60 enrolled patients, with two patients undergoing autopsy evaluations. Four patients did not have neuroimaging and died while on ECMO, without a subsequent autopsy. No enrolled patients had CT scans or MRI performed prior to ECMO cannulation. Twenty-two patients (36.7%) had head ultrasound imaging performed prior to ECMO, with all normal results, and these were performed in infants with congenital heart disease performed as part of standard-of-care serial evaluations in the cardiac ICU. Sixteen patients had brain MRI as their initial neuroimaging, with a median time from ECMO cannulation to imaging of 14.0 days (IQR 7.5–24.0). Seventeen patients underwent head CT as their initial neuroimaging, with a median time from ECMO cannulation to imaging of 5.0 days (IQR 3.5–14.0). Twenty-seven patients underwent head ultrasound imaging as their initial neuroimaging, with a median time from ECMO cannulation to imaging of 1.0 days (IQR 1.0–1.0). Twenty-eight patients (45.1%) had head ultrasounds performed as their exclusive neuroimaging modality.

In total, 19 patients suffered acute brain injury (30.6%), including seven (36.8%) with AIS, four (21.1%) with HS, seven with HIBI (36.9%), and one (5.3%) with both AIS and HIBI. Five patients experienced AIS lateralized to the left hemisphere, and two had AIS lateralized to the right hemisphere. Two patients experienced posterior circulation AIS, and five patients experienced anterior circulation AIS. All four patients with HS had intraventricular hemorrhage (IVH) with bilateral intraparenchymal extension and involvement. All seven patients with HIBI had diffuse involvement. Acute brain injury was recognized on CT in 11 patients (57.9%), on MRI in five patients (26.3%), on head ultrasound imaging in four patients (21.1%), and on postmortem autopsy examinations in two patients (10.5%). Seizures were identified in eight patients (12.9% of the total cohort; 42.1% of the brain injury cohort), all of whom had neuroimaging or autopsy evidence of brain injury. The median time from ECMO cannulation to identification of brain injury via neuroimaging was 7.5 days (IQR 3.5–10.5).

### Physiologic Features Reflecting Brain Injury

Univariate logistic regression demonstrated that acute brain injury was associated with maximal hourly seizure burden (OR = 1.87 [95% CI 1.25–3.77], *p* = 0.021), qEEG SP (OR = 1.06 [95% CI 1.01–1.12], *p* = 0.022), increased interhemispheric differences in qEEG total power (OR = 1.99 [95% CI 1.19–3.97], *p* = 0.023) and amplitude (OR = 2.10 [95% CI 1.21–4.18], *p* = 0.017), and increased differences in TCD TIBI scores between bilateral MCA territories (OR = 3.22 [95% CI 1.40–13.45], *p* = 0.023) (Supplement 4). Multivariate best subset model selection identified that maximal hourly seizure burden (OR = 2.17 [95% CI 1.32–4.14], partial *R*^2^ = 0.49, *p* = 0.011), interhemispheric amplitude differences (OR = 2.41 [95% CI 1.29–5.47], partial *R*^2^ = 0.48, *p* = 0.013), and differences in MCA TIBI scores (OR = 4.66 [95% CI 1.85–20.58], partial *R*^2^ = 0.49, *p* = 0.006) each were independently associated with acute brain injury (area under the receiver operating characteristic curve = 0.92) (Table 2). We did not identify any demographic or ECMO circuit characteristics that were associated with acute brain injury, including for AIS, HS, and HIBI (Supplement 4).

**Table 2:** Independent factors associated with acute brain injury in pediatric ECMO patients

Variables	Multivariable analysis			
	OR (95% CI)	*p* value	Partial *R*^2^	AUROC
Differences in MCA TIBI scores	4.66 (1.85–20.58)	**0.006**	0.49	0.92
Seizure burden	2.07 (1.32–4.14)	**0.011**	0.49	
Amplitude difference	2.41 (1.29–5.47)	**0.013**	0.48	

Bold values represent statistically-significant

*AUROC* area under the receiver operating characteristic curve, *CI* confidence interval, *ECMO* extracorporeal membrane oxygenation, *MCA* middle cerebral artery, *OR* odds ratio, *TIBI* Thrombolysis in Brain Ischemia

### Physiologic Features Associated with AIS

The univariate analysis identified that interictal epileptiform discharges (OR = 13.00 [95% CI 1.23–149.75], *p* = 0.028), differences in bilateral MCA TIBI scores (OR = 3.12 [95% CI 1.44–7.79], *p* = 0.006), increased left MCA flow velocities (OR = 1.04 [95% CI 1.00–1.07], *p* = 0.044), and differences in TCD PI values (OR = 72.66 [95% CI 1.21–5,943.01], *p* = 0.041) were significantly associated with AIS (Supplement 5).

### Physiologic Features Associated with HS

The univariate analysis identified that interhemispheric differences in qEEG alpha power (OR = 133.55 [95% CI 0.94–2,731.40], *p* = 0.046), total power (OR = 3.12 [95% CI 1.57–10.39], *p* = 0.008), and amplitude (OR = 4.18 [95% CI 1.71–19.70], *p* = 0.012) were associated with HS (Supplement 5).

### Physiologic Features Associated with HIBI

The univariate analysis identified increased SP on qEEG (OR = 1.07 [95% CI 1.02–1.14], *p* = 0.010), maximal hourly seizure burden (OR = 1.32 [95% CI 1.09–1.75], *p* = 0.017), the presence of electrographic seizures (OR = 1.32 [95% CI 1.09–1.75], *p* = 0.017), and increased time of ABP below ABPOpt (OR = 1.10 [95% CI 1.02–1.24], *p* = 0.039) as associated with HIBI (Supplement 5).

## Discussion

This study reviewed MMM data from a prospective cohort of pediatric patients who were treated with ECMO. Brain injury was common, with a 30.6% incidence in accordance with prior literature [3, 5, 6, 16, 32–34]. The multivariable analysis revealed that increased maximal hourly seizure burden on cEEG, interhemispheric amplitude differences on qEEG, and differences in MCA TIBI scores using TCD were independently associated acute brain injury with high accuracy. To our knowledge, this is the first prospective study to demonstrate the utility of combining cEEG, qEEG, and TCD monitoring for recognition of brain injury in pediatric ECMO patients.

Continuous electroencephalography (cEEG) has been previously used by various centers to predict outcomes in ECMO patients. In a study by Huang et al., investigators found that electrographic or electroclinical seizures were identified in 40% of patients and were associated with brain injury in infants undergoing ECMO [13]. Electrographic seizure incidence was found to be 18% in another pediatric study [35]. Another retrospective study of 201 pediatric ECMO patients examined EEG features associated with brain injury and found that severely abnormal cEEG background activity and higher EEG seizure burden was associated with mortality. They also reported that the type of ECMO arterial cannulation site (right carotid vs. aorta) correlated with the side of focal cerebral injury, which in 33% is associated with electrographic seizures [18]. qEEG features have not yet been examined as biomarkers detecting acute brain injury in ECMO patients, and our study results suggest that qEEG differences in interhemispheric amplitude may be useful in brain injury recognition.

We have previously identified that alpha and beta power are reduced in regions of anterior circulation in children with AIS not specifically undergoing ECMO support [36]. We did not observe asymmetry of alpha or beta power to be associated with AIS in this study. This difference may relate to the fact that we collected data for both posterior circulation and anterior circulation AIS in this study. Differences in methodological analyses may also explain these differences given that this study investigated whether hemispheric differences in these qEEG characteristics were associated with AIS through logistic regression, whereas the prior study investigated whether there were significant differences in patients who had anterior circulation AIS through dynamic structural equation modeling. This study also included an increased percentage of neonates than our prior study.

Only a few studies have investigated the role of TCD for detecting acute brain injury in pediatric ECMO patients, with none to date demonstrating significant features that accurately detect brain injury. An observational study in the pediatric ECMO population showed that flow velocities during ECMO deviated from age-specific normal values in all major cerebral vessels and across different age groups. This study also reported that global or regional elevations and asymmetries in flow velocity may suggest impending neurologic injury, although statistical significance was not achieved [21]. A multicenter study on TCD evaluation of cerebrovascular physiology in ECMO-supported children found that MCA flow velocities were significantly lower than normative values for critically ill children [20]. However, in accordance with our findings, no significant difference was detected in systolic, diastolic, or mean flow velocities between pediatric patients with and without brain injury. Investigators found that increased PI may be a marker for ischemic injury in young infants on ECMO [20]. We evaluated MCA TIBI grades as a method of extracting the MCA waveform analysis, identifying that those differences in TIBI grades between each MCA territory were independently associated with brain injury in pediatric ECMO patients. Our finding is consistent with previously published literature that revealed a strong association with TIBI grades and brain injury severity among patients with AIS [28].

Regarding model-based indices of CVPR identified from MMM, prior retrospective work reported that ABP was below LLA and above ULA over longer periods of time in patients with acute neurologic events compared with patients without acute neurologic events [15]. We did not observe the same findings in our cohort, although we observed at the univariate level that increased time below ABPOpt was associated with HIBI. Prior work has suggested that greater burden of ABP below ABPOpt (otherwise described as optimal mean arterial blood pressure [MAPopt]) is associated with unfavorable outcomes after pediatric cardiac arrest [28]. Further work is needed to understand whether optimizing ABP toward efficient CVPR may be neuroprotective in nature.

This study is one of few to date investigating the role of combined and integrated MMM toward detection of brain injury among pediatric ECMO patients. Previous studies focused on identifying clinical and laboratory predictors of brain injury in ECMO patients [32] or evaluated specific neuromonitoring modalities without comparison of alternative strategies. We note that we did not identify any clinical or ECMO circuit characteristic as associated with brain injury, highlighting the value of MMM integrating cEEG, qEEG, and TCD.

Our study carried limitations. Sedative agents used in the ICUs at our institution include dexmedetomidine, fentanyl, midazolam, and morphine, which might have some influence on EEG, ABP, and rSO_2_ recordings. Challenges in acquiring neuroimaging for all enrolled patients limited comprehensive and timely detection of brain injury. cEEG was only conducted for certain durations of time during total ECMO monitoring, and its value may not be fully captured as a result. Significant amounts of data were removed because of artifact, which also contributed to limitations regarding missing data. Although MMM provides information in real time, it is not possible to identify the precise occurrence of acute brain injury. We evaluated physiologic activity throughout each patient’s ECMO course, so it remains unclear whether the physiologic observations we identified might reflect underlying brain injury or could be used to rapidly recognize new brain insults during the ECMO course. Not all patients had neuroimaging prior to ECMO cannulation, so we cannot fully exclude that some enrolled patients might have had a prior brain injury not previously recognized. A substantial portion of our patients, primarily neonates, had head ultrasounds performed as their exclusive neuroimaging modality, and this may be limited in fully appraising for evidence of brain injury. To that end, our data were skewed toward an increased population of neonates and infants who may have physiologic characteristics that differ from those of older pediatric patients. We observed relatively low qEEG amplitude output in our analysis, which may partially relate to our skewed age population as well as the quantitative methods of signal processing used to calculate this measure. Although characteristics such as qEEG amplitude asymmetry may relate to brain injury, it remains unclear whether such changes can be visualized by clinicians at the bedside or whether such information may be better suited for patient alarm systems that account for such information toward improved recognition of brain injury. We did not prospectively examine how changes in the neurologic examination identify brain injury any differently from our neurologic monitoring modalities, so we are limited regarding our ability to extrapolate the degree to which MMM might provide added benefit as compared to serial neurologic examinations. Our sample size was insufficient for age stratification of our multivariable model or for multivariable analysis of specific causes of acute brain injury, including AIS, HS, and HIBI. A larger sample size arising from a multicenter cohort may be helpful for such investigations. Future work focused on specific age cohorts, specific forms of ECMO support, and similar underlying disease etiologies are needed to better understand how these results may relate to such populations. We excluded microhemorrhages, extra-axial fluid collections, and small white matter hyperintensities as markers of acute brain injury, but the cumulative contribution of these elements may relate to outcomes and warrants further investigation in future studies.

## Conclusions

Acute brain injury is a common complication in pediatric patients undergoing ECMO support. Higher seizure burden, interhemispheric qEEG amplitude differences, and TCD MCA TIBI score differences are independently associated with brain injury in children requiring ECMO. Detection of brain injury through MMM that incorporates cEEG, qEEG, and TCD may provide an opportunity for accurate recognition of injury, with potential opportunities to offer neuroprotective strategies of care.

### Supplementary Information

Below is the link to the electronic supplementary material.

Supplementary file1 (DOCX 47 KB)
