# Supplementary material for: Neurophysiologic Features Reflecting Brain Injury During Pediatric ECMO Support
Source: Neurocrit Care. 2023 Sep 11;40(2):759–68. doi: 10.1007/s12028-023-01836-9 (PMC10959789; doi:10.1007/s12028-023-01836-9)
Supplement: Supplementary file 1 — Supplementary file1 (DOCX 47 KB) [file 12028_2023_1836_MOESM1_ESM.docx]

**Supplement 1. Evaluating for optimal cerebrovascular pressure reactivity based upon cerebral oximetry index**

To account for potential clinically actionable biomarkers extracted from the cerebral oximetry index (COx), we evaluated the % time that patients had ABP values below the lower limit of cerebrovascular pressure reactivity (LLA) or above the upper limit of cerebrovascular pressure reactivity (ULA), as well as optimal blood pressure for cerebrovascular pressure reactivity (ABPOpt). ABPOpt values were identified using previously described methods of plotting ABP values for COx and identifying the minimum ABP value on a U-shaped parabolic curve fit to data with a multiwindow algorithm. The multiwindow algorithm used a maximum calculation window of 28,800 seconds and minimum calculation period of 7200 seconds, with steps of 600 seconds. This develops 37 averaged ABPOpt values for 8 hours of monitoring. This averaged value is updated every minute, meaning that the computed value in time series consists of an average of 36 ABPOpt values. ABPOpt values were taken from a moving 4-hour window. ABPOpt values were rejected in cases of an unreliable U-shaped ABPOpt value. As in prior work, we implemented five curve fitting criteria to keep or remove the curve for building an ABPOpt time series, which included that (1) each ABP bin must be at least 1% of the total count data, (2) at least 50% of the data in the window must be included in the curve fit, (3) variation in COx of 0.2 is mandated, (4) the COx range of interest is enforced to be between – 0.3 and 0.6, and (5) the coefficient of determination of the fitted R^2^ value must be at least 0.0. LLA and ULA are determined for COx as ABP values at which the curve crossed threshold values of 0.3, represented impaired cerebrovascular pressure reactivity as used or suggested in previous studies. LLA and ULA were determined as the lowest and highest ABP value at 0.3, respectively. The % time of ABP values below LLA and above ULA, as well as the % time below ABPOpt were calculated for each patient during their ECMO duration.

**Supplement 2. Model based indices of cerebrovascular pressure reactivity and autonomic function**

| **Metric** | **Definition** | **Signals considered** | **Calculation** | **Interpretation** |
| --- | --- | --- | --- | --- |
| *Cerebral autoregulation index (higher=impaired)* | | | | |
| COx | Cerebral oximetry index | ABP, rSO2 | Correlation coefficient between ABP and rSO2 | Higher COx=impaired autoregulation |
| *Autonomic function indices (lower=impaired)* | | | | |
| BRs | Baroreflex sensitivity | ECG, ABP | Modified cross-correlation method  of R–R intervals and systolic blood  pressure | Lower BRS = impaired autonomic  function |
| HRsd | Standard deviation of HR | ECG | Standard deviation of R–R intervals | Lower HRsd = impaired autonomic  function |
| HRrmssd | Root-mean-square of successive  differences | ECG | Root-mean-square of the standard deviation of R–R intervals | Lower HRrmssd = impaired autonomic  function |
| LHF | Low–high frequency ratio | ECG | Spectral power in R–R low frequency  (0.04–0.15 Hz) divided by high frequency (0.15–0.4 Hz) | Lower LHF = impaired autonomic function |

Abbreviations: ABP, arterial blood pressure; BRS, baroreflex sensitivity; COx, cerebral oximetry index; ECG, electrocardiogram; HRsd, standard deviation of R-R intervals; HRrmssd, root mean square of successive differences of R-R intervals; Hz, hertz; LHF, low frequency–high frequency ratio.

**Supplement 3. Subject Enrollment**

The study cohort was identified based upon daily screening of all patients admitted to the pediatric neonatal, cardiac, or pediatric intensive care units by a research coordinator that was available to enroll patients around the clock on all days of the year. The screening procedure involved assessment of the admission diagnosis and notification of the ECMO team regarding new patients who underwent cannulation. Logistical reasons to exclude patients included when the research team was not available (e.g., vacation), family not available to give consent, and early decisions of withdrawal of life-sustaining therapies at time of screening.

**Supplement 4. Univariate analysis of factors associated with acute brain injury in pediatric ECMO patients**

|  | **Univariate analysis** | | |
| --- | --- | --- | --- |
| **Variable** | **OR** | **95% CI** | ***p* value** |
| Age | 0.95 | 0.84 – 1.05 | 0.342 |
| Female vs. male | 1.19 | 0.38 – 3.77 | 0.764 |
| **ECMO Characteristics** |  |  |  |
| Circuit VA vs. VV | 2.47 | 0.57 – 17.21 | 0.274 |
| Venous cannula size | 1.00 | 0.91 – 1.09 | 0.976 |
| Arterial cannula size | 0.98 | 0.83 – 1.13 | 0.798 |
| Length of hospital stay, days | 1.00 | 0.99 – 1.01 | 0.441 |
| ECMO duration, days | 1.03 | 0.97 - 1.11 | 0.329 |
| Pump flow rate, max (lpm) | 0.82 | 0.59-1.16 | 0.266 |
| Pump flow rate, min (lpm) | 0.61 | 0.34-1.12 | 0.109 |
| Sweep gas flow (O_2_), max (lpm) | 1.07 | 0.90-1.27 | 0.456 |
| Sweep gas flow (O_2_), min (lpm) | 0.92 | 0.52-1.61 | 0.759 |
| FsO_2_, max (%) | 0.99 | 0.96-1.02 | 0.377 |
| FsO_2_, min (%) | 0.99 | 0.97-1.01 | 0.331 |
| Anti Xa level (maximum) | 1.13 | 0.68-1.87 | 0.621 |
| Anti Xa level (minimum) | 1.98 | 0.02-169.05 | 0.764 |
| E-CPR prior to ECMO canulation | 2.64 | 0.77-9.04 | 0.116 |
| **EEG Characteristics** |  |  |  |
| Interictal epileptic discharge presence | 2.50 | 0.27 – 22.87 | 0.386 |
| Interhemispheric differences in alpha power | 10.57 | 0.48 – 267.29 | 0.132 |
| Interhemispheric differences in beta power | 0.99 | 0.30 – 2.33 | 0.988 |
| Interhemispheric differences in theta power | 0.92 | 0.59 – 1.10 | 0.513 |
| Interhemispheric differences in delta power | 1.00 | 0.83 – 1.14 | 0.995 |
| Interhemispheric differences in total power | 1.99 | 1.19 – 3.97 | 0.023 |
| Interhemispheric differences in ADR | 0.75 | 0.26 – 1.23 | 0.576 |
| Interhemispheric differences in amplitude, µV | 2.10 | 1.21 – 4.18 | **0.017** |
| Average alpha power | 0.32 | 0.01 – 6.09 | 0.479 |
| Average beta power | 0.30 | 0.19 – 2.38 | 0.339 |
| Average theta power | 0.57 | 0.22 – 0.99 | 0.180 |
| Average delta power | 0.88 | 0.27 – 1.95 | 0.124 |
| Average total power | 0.93 | 0.73 – 1.01 | 0.306 |
| Average alpha-delta power ratio | 4.71 | 0.10 – 213.9 | 0.416 |
| Average amplitude, µV | 0.89 | 0.74 – 1.03 | 0.164 |
| Suppression percentage, % | 1.06 | 1.01 – 1.12 | **0.022** |
| Maximal hourly seizure burden | 1.87 | 1.25 – 3.77 | **0.021** |
| **TCD Characteristics** |  |  |  |
| L MCA FV | 1.01 | 0.99 – 1.05 | 0.207 |
| L MCA PI | 0.61 | 0.15 – 2.23 | 0.469 |
| R MCA FV | 1.00 | 0.96 – 1.03 | 0.903 |
| R MCA PI | 0.38 | 0.09 – 1.36 | 0.160 |
| Mean FV | 1.01 | 0.98 – 1.05 | 0.474 |
| Mean PI | 0.45 | 0.10 – 1.69 | 0.256 |
| Differences in FV | 7.45 | 0.40 – 164.59 | 0.174 |
| Differences in PI | 5.11 | 0.18 – 139.39 | 0.326 |
| Differences in MCA TIBI scores | 3.22 | 1.40 – 13.4513.45 | 0.023 |
| **Cerebral Regional Oximetry Characteristics** |  |  |  |
| RSO_2_ median | 0.98 | 0.94 – 1.02 | 0.394 |
| ABPOpt median | 0.98 | 0.93 – 1.02 | 0.290 |
| COx median | 980.06 | 1.21 – 1824621.25 | 0.054 |
| Percent of time spent above ABPOpt | 0.98 | 0.94 – 1.02 | 0.294 |
| Percent of time spent above ULA | 1.00 | 0.87 – 1.15 | 0.947 |
| Percent of time spent below ABPOpt | 1.03 | 0.98 – 1.08 | 0.290 |
| Percent of time spent below LLA | 1.01 | 0.99 – 1.03 | 0.373 |
| **Autonomic Function Characteristics** |  |  |  |
| LHFRatio median | 1.07 | 0.70 – 1.57 | 0.747 |
| HRrmssd median | 0.95 | 0.85 – 1.03 | 0.288 |
| HRsd median | 0.74 | 0.42 – 1.11 | 0.218 |
| BRs median | 0.93 | 0.77 – 1.00 | 0.406 |
| Other Physiologic Characteristics |  |  |  |
| CVP median | 0.93 | 0.74 – 1.01 | 0.339 |
| SpO_2_ median | 1.05 | 0.92 – 1.26 | 0.506 |

Abbreviations: ABPOpt, optimal arterial blood pressure; ADR, alpha delta power ratio; BRs, baroreflex sensitivity index; COx, cerebral oximetry index; CVP, central venous pressure; FV, flow velocity; HRrmssd, root-mean-square of successive differences of R-R intervals; HRsd, Standard deviation of R-R intervals; LHF Ratio, Low to high frequency; LLA, lower limit of cerebrovascular pressure reactivity; lpm, liters per minute; MCA, middle cerebral artery; PI, pulsatility index; RSO_2_, regional oxygen saturation; SpO_2_, pulse oxygen saturation; TIBI, Thrombolysis in Brain Ischemia system; ULA, upper limit of cerebrovascular pressure reactivity; V-A, veno-arterial; V-V, veno-venous; y, years; %, percentage; E-CPR, extracorporeal cardiopulmonary resuscitation, µV, microvolts.

**Supplement 5. Factors associated with each brain injury subset in pediatric ECMO patients**

|  | **AIS** | | **HS** | | **HIBI** | |
| --- | --- | --- | --- | --- | --- | --- |
| **Variable** | **OR (95% CI)** | ***p* value** | **OR (95% CI)** | ***p* value** | **OR (95% CI)** | ***p* value** |
| Age, years | 0.99 (0.84-1.12) | 0.857 | 1.03 (0.82-1.22) | 0.743 | 0.84 (0.57-1.01) | 0.170 |
| Age < 1 y vs. age ≥1 y | 1.54 (0.30-11.46) | 0.622 | 1.19 (0.11-26.46) | 0.890 | 0.54 (0.12-2.53) | 0.423 |
| Male vs. female sex | 1.19 (0.24-6.54) | 0.826 | 0.42 (0.02-4.64) | 0.491 | 0.48 (0.09-2.16) | 0.347 |
| Circuit VA vs. VV | 1.86 (0.14-25.15) | 0.995 | 1.77 (0.08-19.93) | 0.652 | 2.22 (0.35-43.50) | 0.475 |
| Venous cannula size | 1.03 (0.91-1.16) | 0.599 | 1.09 (0.92-1.31) | 0.288 | 0.94 (0.81-1.07) | 0.412 |
| Arterial cannula size | 1.13 (0.94-1.35) | 0.180 | 0.00 (0.00-5E35) | 0.997 | 0.92 (0.71-1.11) | 0.433 |
| Length of hospital stay, days | 0.99 (0.96-1.01) | 0.426 | 0.98 (0.91-1.01) | 0.506 | 1.00 (0.98-1.01) | 0.802 |
| ECMO duration, days | 0.98 (0.86-1.08) | 0.755 | 1.02 (0.85-1.15) | 0.768 | 0.93 (0.78-1.04) | 0.274 |
| Electroencephalographic seizure presence | 1.14 (0.96-1.37) | 0.113 | 2.62 (0.14-29.36) | 0.427 | 1.32 (1.09-1.75) | **0.017** |
| Interictal epileptic discharge presence | 13.00 (1.23-149.75) | **0.028** | 0.00 (0.00-8E186) | 0.996 | 0.00 (0.00-7E90) | 0.999 |
| Interhemispheric differences in alpha power | 1.75 (0.01-82.33) | 0.794 | 133.55 (0.94-2731.40) | **0.046** | 1.13 (0.01-49.30) | 0.955 |
| Interhemispheric differences in beta power | 1.24 (0.28-3.17) | 0.686 | 1.11 (0.04-3.70) | 0.899 | 0.79 (0.06-2.33) | 0.755 |
| Interhemispheric differences in theta power | 0.95 (0.50-1.16) | 0.755 | 0.96 (0.31-1.22) | 0.860 | 0.91 (0.38-1.14) | 0.638 |
| Interhemispheric differences in delta power | 0.96 (0.59-1.14) | 0.755 | 1.07 (0.82-1.25) | 0.421 | 0.96 (0.61-1.13) | 0.735 |
| Interhemispheric differences in total power | 0.87 (0.20-1.38) | 0.755 | 3.12 (1.57-10.39) | **0.008** | 1.18 (0.61-1.94) | 0.549 |
| Interhemispheric differences in ADR | 1.43 (0.93-2.20) | 0.103 | 0.58 (0.00-1.43) | 0.839 | 0.54 (0.00-1.26) | 0.741 |
| Interhemispheric differences in amplitude, µV | 0.76 (0.20-1.62) | 0.586 | 4.18 (1.71-19.70) | **0.012** | 1.27 (0.64-2.27) | 0.426 |
| Average alpha power | 0.04 (0.00-4.27) | 0.248 | 17.67 (0.09-2328.41) | 0.230 | 0.02 (0.00-1.77) | 0.137 |
| Average beta power | 0.25 (0.00-4.51) | 0.462 | 1.63 (0.01-26.90) | 0.778 | 0.06 (0.00-1.95) | 0.203 |
| Average theta power | 0.75 (0.23-1.17) | 0.526 | 0.77 (0.12-1.22) | 0.692 | 0.45 (0.11-1.01) | 0.207 |
| Average delta power | 0.86 (0.61-1.05) | 0.238 | 1.03 (0.78-1.24) | 0.792 | 0.75 (0.49-0.98) | 0.100 |
| Average TP | 0.87 (0.64-1.07) | 0.252 | 1.08 (0.83-1.36) | 0.508 | 0.83 (0.62-1.03) | 0.158 |
| Average ADR | 1.40 (0.00-203.90) | 0.899 | 0.04 (0.00-122.78) | 0.506 | 8.25 (0.06-939.65) | 0.376 |
| Average AMP, µV | 0.81 (0.56-1.03) | 0.161 | 1.07 (0.81-1.36) | 0.572 | 0.77 (0.53-0.99) | 0.095 |
| Suppression percentage, % | 1.02 (0.97-1.07) | 0.382 | 1.04 (0.98-1.10) | 0.149 | 1.07 (1.02-1.14) | **0.010** |
| Maximal hourly seizure burden | 1.14 (0.96-1.37) | 0.113 | 0.00 (0.00-7E98) | 0.996 | 1.32 (1.09-1.75) | **0.017** |
| L MCA FV | 1.04 (1.00-1.07) | **0.044** | 1.02 (0.96-1.07) | 0.515 | 1.00 (0.97-1.04) | 0.786 |
| L MCA PI | 0.92 (0.14-4.97) | 0.922 | 1.03 (0.06-11.85) | 0.982 | 0.17 (0.01-1.24) | 0.116 |
| R MCA FV | 1.00 (0.96-1.05) | 0.842 | 0.92 (0.82-1.01) | 0.134 | 1.00 (0.96-1.04) | 0.753 |
| R MCA PI | 0.20 (0.02-1.23) | 0.118 | 1.75 (0.15-18.64) | 0.633 | 0.59 (0.09-3.03) | 0.551 |
| Mean FV | 1.03 (0.99-1.08) | 0.170 | 0.99 (0.91-1.05) | 0.754 | 1.01 (0.96-1.05) | 0.748 |
| Mean PI | 0.41 (0.05-2.44) | 0.358 | 1.39 (0.09-16.11) | 0.793 | 0.32 (0.03-2.00) | 0.259 |
| Differences in FV | 3.84 (0.07-116.49) | 0.454 | 18.25 (0.13-1449.68) | 0.182 | 6.21 (0.14-189.11) | 0.294 |
| Differences in PI | 72.66 (1.21-5943.01) | **0.041** | 1.31 (0.00-485.49) | 0.936 | 3.50 (0.03-212.61) | 0.561 |
| Differences in TIBI scores | 3.12 (1.44-7.79) | **0.006** | 2.20 (0.72-5.65) | 0.098 | 1.36 (0.46-2.89) | 0.497 |
| ABPOpt median | 1.05 (0.98-1.12) | 0.171 | 0.96 (0.79-1.08) | 0.594 | 1.01 (0.93-1.09) | 0.727 |
| RSO_2_ median | 1.03 (0.96-1.12) | 0.495 | 0.94 (0.88-1.01) | 0.062 | 1.00 (0.95-1.07) | 0.943 |
| COx median | 23831.72 (2.68-9345428.03) | **0.039** | 0.25 (0.00-95003.70) | 0.836 | 9.07 (0.00-65925.04) | 0.621 |
| LHFRatio median | 0.96 (0.48-1.59) | 0.881 | 1.43 (0.70-2.61) | 0.243 | 0.86 (0.40-1.48) | 0.633 |
| HRrmssd median | 0.87 (0.65-1.02) | 0.202 | 1.06 (0.92-1.17) | 0.321 | 0.94 (0.77-1.05) | 0.413 |
| HRsd median | 0.51 (0.16-1.07) | 0.160 | 1.13 (0.52-1.82) | 0.662 | 0.64 (0.24-1.19) | 0.278 |
| BRs median | 0.78 (0.50-0.97) | 0.190 | 1.00 (0.03-1.01) | 0.874 | 0.92 (0.67-1.00) | 0.507 |
| SPO_2_ median | 1.37 (0.98-2.42) | 0.187 | 1.03 (0.83-1.65) | 0.833 | 0.97 (0.84-1.19) | 0.708 |
| CVP median | 0.69 (0.40-0.96) | 0.101 | 1.06 (0.92-1.74) | 0.772 | 1.07 (0.94-1.49) | 0.625 |
| Percent of time spent above ABPOpt | 1.01 (0.95-1.07) | 0.799 | 1.00 (0.92-1.09) | 0.937 | 0.91 (0.81-0.98) | **0.039** |
| Percent of time spent above ULA | 1.09 (0.90-1.28) | 0.336 | 1.00 (0.69-1.28) | 0.984 | 0.85 (0.56-1.08) | 0.283 |
| Percent of time spent below ABPOpt | 0.99 (0.94-1.06) | 0.799 | 0.99 (0.91-1.09) | 0.841 | 1.10 (1.02-1.24) | **0.039** |
| Percent of time spent below LLA | 0.99 (0.96-1.03) | 0.736 | 0.99 (0.94-1.04) | 0.792 | 1.03 (1.00-1.08) | 0.080 |

Abbreviations: AB*P*Opt, optimal arterial blood pressure; ADR, alpha delta power ratio; BRs, baroreflex sensitivity index; Cox, cerebral oximetry index; CVP, central venous pressure; FV, flow velocity; HRrmssd, hear rate root-mean-square of successive differences; HRsd, Standard deviation of heart rate; LHF Ratio, Low to high frequency; LLA, lower limit of autoregulation; MCA, middle cerebral artery; PI, pulsatility ind-ex; RSO_2_, regional oxygen saturation; SpO_2_, pulse oxygen saturation; TIBI, Thrombolysis in Brain Ischemia system; ULA, upper limit of autoregulation, µV, microvolts; %, percentage.

**Supplement 6: Electroencephalographic and physiologic characteristics of patients on ECMO support**

|  | **Median (IQR)** | **Range** |
| --- | --- | --- |
| Interhemispheric differences in alpha power | 0.08 (0.03, 0.23) | 0.00, 0.74 |
| Interhemispheric differences in beta power | 0.05 (0.02, 0.14) | 0.00, 3.75 |
| Interhemispheric differences in theta power | 0.15 (0.03, 0.34) | 0.00, 21.88 |
| Interhemispheric differences in delta power | 0.41 (0.23, 1.48) | 0.00, 26.70 |
| Interhemispheric differences in total power | 0.38 (0.20, 1.01) | 0.00, 6.02 |
| Interhemispheric differences in ADR | 0.01 (0.00, 0.02) | 0.00, 11.63 |
| Interhemispheric differences in amplitude, µV | 0.51 (0.20, 1.13) | 0.00, 5.58 |
| Average alpha power | 0.37 (0.27, 0.48) | 0.02, 1.15 |
| Average beta power | 0.43 (0.33, 0.56) | 0.03, 1.90 |
| Average theta power | 1.47 (0.96, 1.83) | 0.05, 24.32 |
| Average delta power | 4.31 (0.10, 25.06) | 0.10, 25.06 |
| Average TP | 4.04 (2.56, 7.67) | 0.07, 19.17 |
| Average ADR | 0.23 (0.13, 0.36) | 0.02, 0.71 |
| Average AMP, µV | 4.30 (3.14, 8.51) | 0.08, 19.20 |
| Suppression percentage, % | 1.95 (0.05, 12.82) | 0.00, 63.02 |
| Maximal hourly seizure burden, all patients (n=62) | 0.00 (0.00, 0.00) | 0.00, 20.00 |
| Maximal hourly seizure burden, patients with brain injury (n=19) | 0.00 (0.00, 6.00) | 0.00, 20.00 |
| Maximal hourly seizure burden, patients without patients (n=43) | 0.00 (0.00, 0.00) | 0.00, 0.00 |
| L MCA FV, cm/sec | 44.10 (34.23, 59.45) | 10.00, 113.00 |
| L MCA PI | 0.86 (0.61, 1.30) | 0.15, 2.14 |
| R MCA FV, cm/sec | 43.45 (35.25, 51.98) | 12.00, 97.80 |
| R MCA PI | 0.85 (0.62, 1.30) | 0.10, 2.13 |
| Mean FV, cm/sec | 47.48 (36.88, 56.75) | 11.00, 97.40 |
| Mean PI | 0.83 (0.59, 1.26) | 0.13, 2.14 |
| Differences in FV, cm/sec | 1.6 (0.80, 3.02) | 0.00, 10.00 |
| Differences in PI | 0.15 (0.05, 0.27) | 0.00, 0.68 |
| Differences in TIBI scores | 0.00 (0.00, 0.00) | 0.00, 3.00 |
| ABPOpt median, mmHg | 67.25 (59.09, 72.50) | 51.42, 103.20 |
| RSO_2_ median, % | 67.79 (61.43, 76.80) | 15.00, 89.00 |
| COx median | 0.02 (-0.02, 0.07) | -0.18, 0.25 |
| LHFRatio median | 1.59 (0.76, 2.42) | 0.06, 7.57 |
| HRrmssd median | 5.65 (3.43, 10.90) | 0.91, 37.24 |
| HRsd median | 1.77 (1.41, 2.77) | 0.34, 10.68 |
| BRs median | 4.39 (2.93, 7.06) | 1.10, 605.20 |
| SpO_2_ median | 96.25 (95.00, 97.92) | 76.16, 100.00 |
| CVP median, mmHg | 12.88 (9.85, 14.48) | 2.00, 19.60 |
| Percent of time spent above ABPOpt | 18.38 (7.23, 26.03) | 0.00, 48.98 |
| Percent of time spent above ULA | 1.62 (0.44, 5.65) | 0.00, 15.22 |
| Percent of time spent below ABPOpt | 81.62 (73.97, 92.77) | 51.02, 100.00 |
| Percent of time spent below LLA | 59.34 (41.40, 76.30) | 11.65, 100.00 |

Abbreviations: ECMO, extracorporeal membrane oxygenation; IQR, interquartile range; ADR, alpha-delta power ratio; TP, total power; AMP, amplitude; L, left; MCA, middle cerebral artery; PI, pulsatility index; R, right; TIBI, thrombolysis in brain ischemia; ABPOpt, optimal arterial blood pressure; mmHg, millimeters of mercury; RSO_2_, cerebral regional oximetry; COx, cerebral oximetry index; LHFRatio, low-high frequency ratio; HRrmssd, root mean square standard deviation of R-R intervals; HRsd, standard deviation of R-R intervals; BRs, baroreflex sensitivity; SpO_2_, peripheral oxygen saturation; CVP, central venous pressure; ULA, upper limit of autoregulation; LLA, lower limit of autoregulation; %, percentage; µV, microvolts, n, count.
